# Foliar application of sodium selenate induces regulation in yield formation, grain quality characters and 2-acetyl-1-pyrroline biosynthesis in fragrant rice

**DOI:** 10.1186/s12870-019-2104-4

**Published:** 2019-11-15

**Authors:** Haowen Luo, Bin Du, Longxin He, Axiang Zheng, Shenggang Pan, Xiangru Tang

**Affiliations:** 1 Department of Crop Science and Technology, College of Agriculture, South China Agricultural University, Guangzhou, 510642 China; 2 Scientific Observing and Experimental Station of Crop Cultivation in South China, Ministry of Agriculture, Guangzhou, 510642 China

**Keywords:** Fragrant rice, 2-acetyl-1-pyrroline, Sodium selenate, Yield, Grain quality

## Abstract

**Background:**

Selenium (Se) is a beneficial element for higher plants and essential for mammals. To study the effect of the foliar application of sodium selenate on fragrant rice performance, a pot experiment was conducted in Guangdong, China. At the initial heading stage, one-time foliar application of sodium selenate with concentrations of 0, 10, 20, 30, 40 and 50 μmol·L^− 1^ (named CK, Se1, Se2, Se3, Se4 and Se5, respectively) were foliar applied on two fragrant rice varieties, ‘*Meixiangzhan-2*’ and ‘*Xiangyaxiangzhan*’.

**Results:**

Selenate application at the initial heading stage not only improved the grain yield of fragrant rice by increasing the seed-setting rate and grain weight, but also promoted the grain quality by increasing crude protein contents and lowering the chalky rice rate. Furthermore, Se applications enhanced the biosynthesis of 2-acetyl-1- pyrroline (2-AP), the main aromatic compound, by increasing the contents of precursors (△1- pyrroline, proline and pyrroline-5-carboxylic acid (P5C)) and the activities of enzymes (proline dehydrogenase (PRODH), △1-pyrroline-5-carboxylic acid synthetase (P5CS), and ornithine aminotransferase (OAT)) in fragrant rice. The results also showed that foliar application of sodium selenate enhanced the antioxidant system of both varieties by promoting the activities of peroxidase (POD), superoxide dismutase (SOD), catalase (CAT) and reducing the contents of malondialdehyde (MDA). Furthermore, the real-time PCR analyses depicted that foliar application of selenate up-regulated the *GPX1*, *GPX4* and *CATC* transcripts. The higher antioxidative enzymatic activities might strength the stress resistant to ensure the stability of yield in fragrant rice form abiotic stress.

**Conclusions:**

Foliar applications of sodium selenate at the initial heading stage increased the grain 2-AP content by enhancing the biosynthesis-related enzymes and precursors. The grain yield and quality of fragrant rice also increased due to selenate application. Furthermore, foliar application of selenate promoted the activities of enzymes such as POD, SOD and CAT and up-regulated the expression of gene *GPX4*, *GPX1* and *CATC*.

## Background

Fragrant rice is a specialty rice which desired by people because of its good taste and special aroma [1]. The unique aroma is the most notable character of fragrant rice. Previous study showed that the volatile compounds in the fragrance are much complicated while there was more than 200 volatile compounds were detected in instrumental analyses [2]. Maraval [3] further pointed out that 2-acetyl-1-pyrroline is mainly responsible for aromatic character of fragrant rice and this point is widely accepted by the world recently. In last decades, the price of aromatic rice has greatly increased in the markets just like the demand for aromatic rice [4], therefore, the fragrant rice production draw more interests from farmers and experts because of the great benefits.

**Fig. 1:**
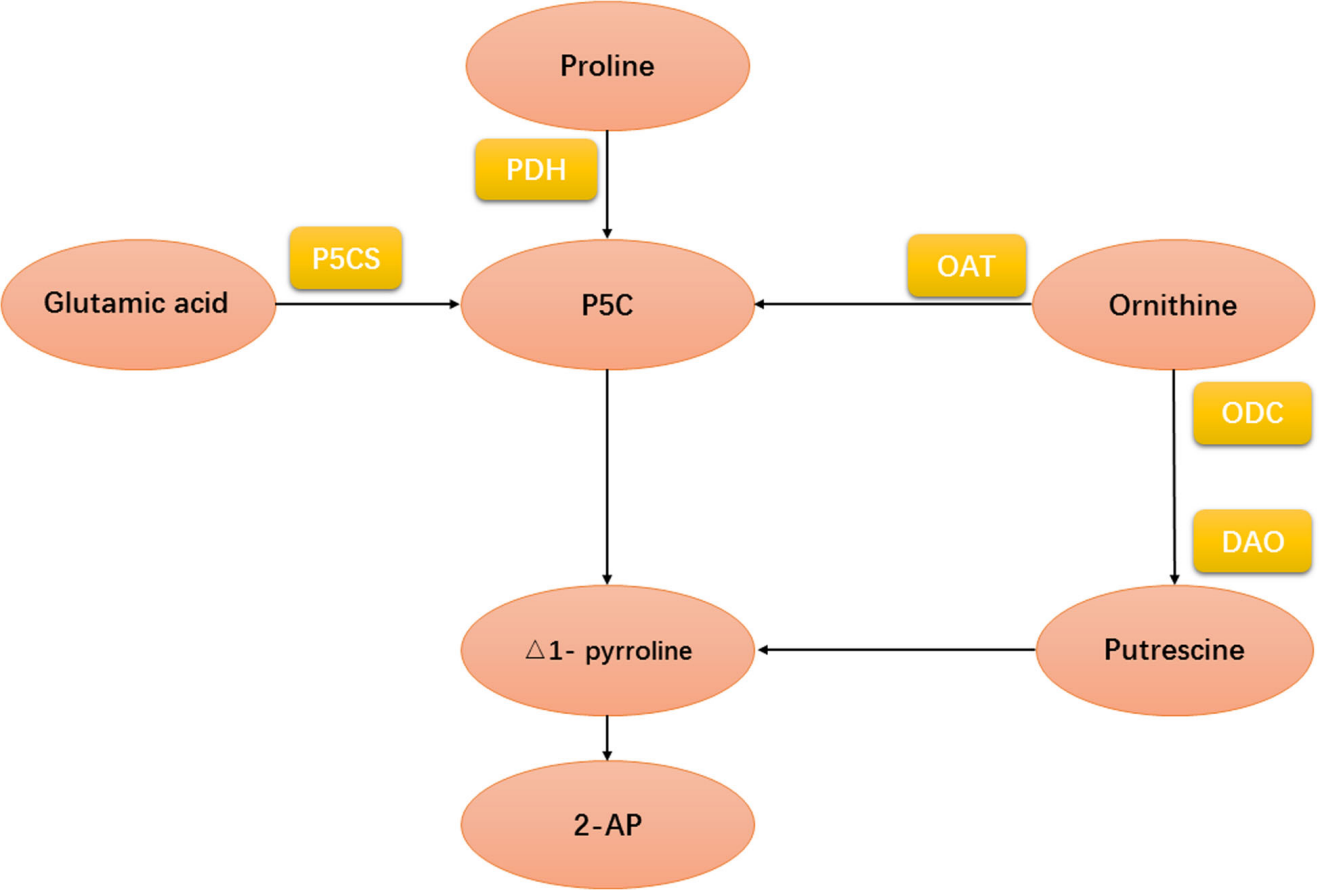
Possible mechanism of 2-AP formation in fragrant rice

As the key component of fragrance, the 2-AP biosynthesis in fragrant rice was a very complicated phenomenon. Early researches have shown some precursors and enzymes in the pathways of 2-AP biosynthesis. For example, in 1993, Seitz [5] demonstrated that proline was the most important precursor and might directly involve in 2-AP formation. Previous study also indicated that activities of △1 pyrroline-5-carboxylic acid synthetase (P5CS) and ornithine aminotransferase (OAT) had positive correlations with 2-AP while the pyrroline-5-carboxylic acid (P5C) was also involved in 2-AP biosynthesis in aromatic rice varieties [6]. Some early studies even indicated several important steps in 2-AP biosynthesis in fragrant rice and the possible biosynthesis pathway of 2-AP was depicted in Fig. 1 [7–9].

Lately, many studies have discovered some agronomy strategies and managements could improve the 2-AP concentrations in aromatic rice. The study of Deng [10] showed that the interaction of water management and extra nitrogen application during the grain filling phase significant increased the grain 2-AP content in fragrant rice. Bao [8] demonstrated that the water management of alternation of wetting and drying during the grain filling stage greatly enhanced the biosynthesis of 2-AP content in fragrant rice. Furthermore, Mo [11] and Li [12] indicated that s silicon (Si) and manganese (Mn) also could induced regulation in 2-AP biosynthesis in fragrant rice, respectively. Therefore, foliar application of microelement could be a new method to improved 2-AP content in fragrant rice.

Selenium (Se) is not only an essential element for humans and animals, but also a beneficial element for higher plants. An early study demonstrated that Se is a necessary chemical element for human body while the Se deficiency can lead to low immune function, increased risk of death and decreased cognitive ability [13]. The study of Vinceti [14] revealed that coronary heart disease, hypertension, Kashin-Beck disease and other diseases are all related to selenium deficiency while the low selenium environment is one of the main factors for the occurrence of these diseases. Previous study already showed that Se has positive influences in growth and development of many crops. For example, the research of He [15] revealed that application of Selenite application at rupturing stage significantly increased the grain yield of rice and promoted the activities of peroxidase (POD), superoxide dismutase (SOD) and catalase (CAT) in leaves. Lai [16] demonstrated that that exogenous Se applications could increase the rice yield by regulating the photosynthesis. Furthermore, Wang [17] indicated that lower Se treatments could activate antioxidant system and enhance photosynthesis while higher Se treatment damaged photosynthesis apparatus and inhibited photosynthesis. Therefore, Se has potential to be the exogenous regulator in fragrant rice production.

Until now, there is still no any report about the effect of Se on fragrant rice performance such as antioxidant system and 2-AP biosynthesis. Hence, this study was conducted in Guangdong province (major rice producing province in South China) with the hypothesis that foliar of sodium selenate can be used to regulated the 2-AP biosynthesis and other physiological characteristics in fragrant rice.

## Results

### 2-AP content in grains

**Fig. 2:**
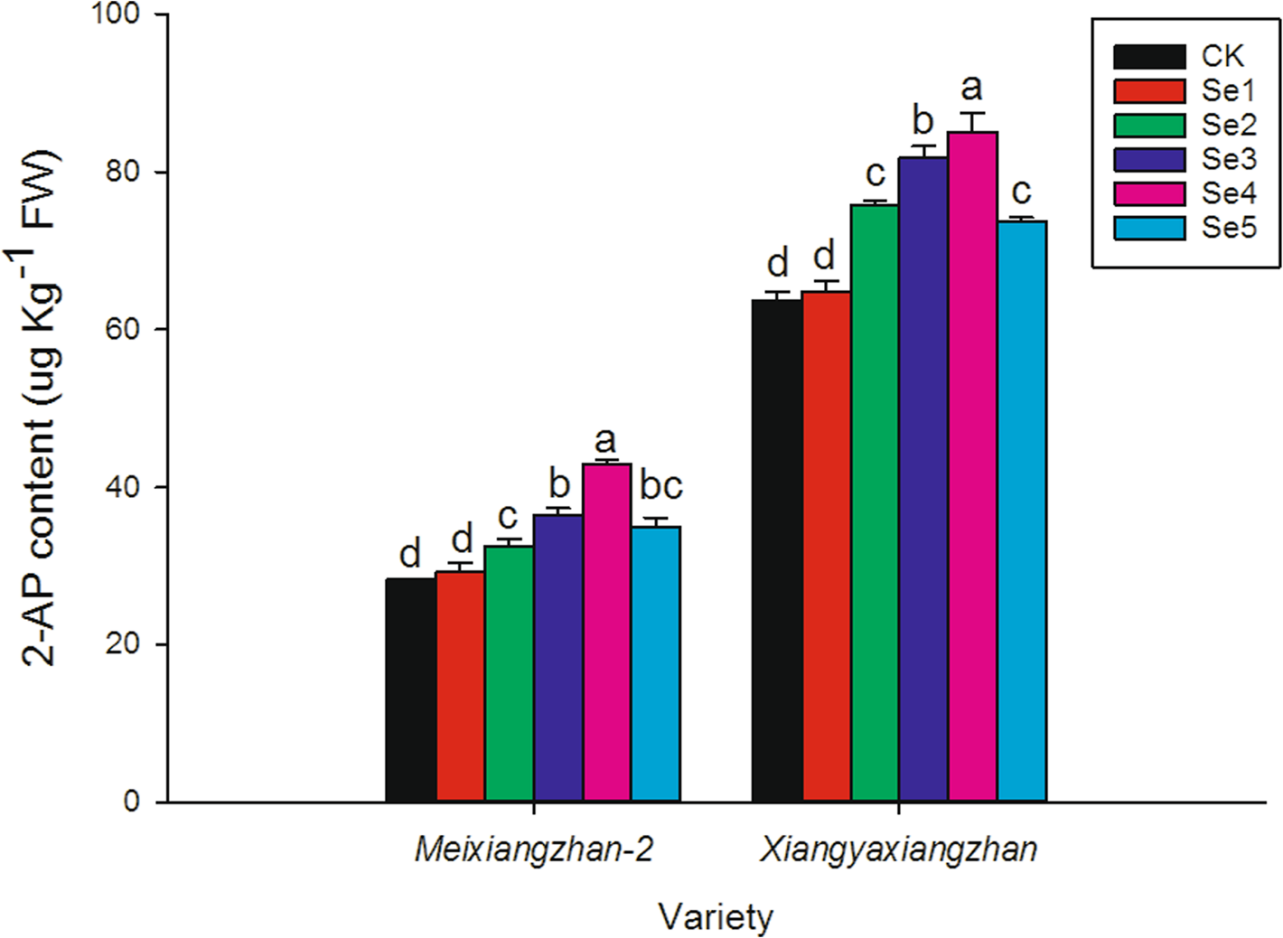
Effect of sodium selenate on the contents of 2-AP in grains Means sharing a common letter do not differ significantly at (*P* ≤ 0.05) according to the LSD test for both varieties. The same as below

As shown in Fig. 2, foliar application of sodium selenate significantly affected the 2-AP content in grains. For *Meixiangzhan-2*, 15.27, 29.25, 52.03 and 24.07% higher grain 2-AP contents were recorded in Se2, Se3, Se4 and Se5 than CK, respectively. For *Xiangyaxiangzhan*, compared to CK, Se2, Se3, Se4 and Se5 increased the 2-AP content by 18.94, 28.54, 33.63 and 15.84%, respectively.

### Synthetic precursors and enzymes involved in 2-AP biosynthesis in grains

**Fig. 3:**
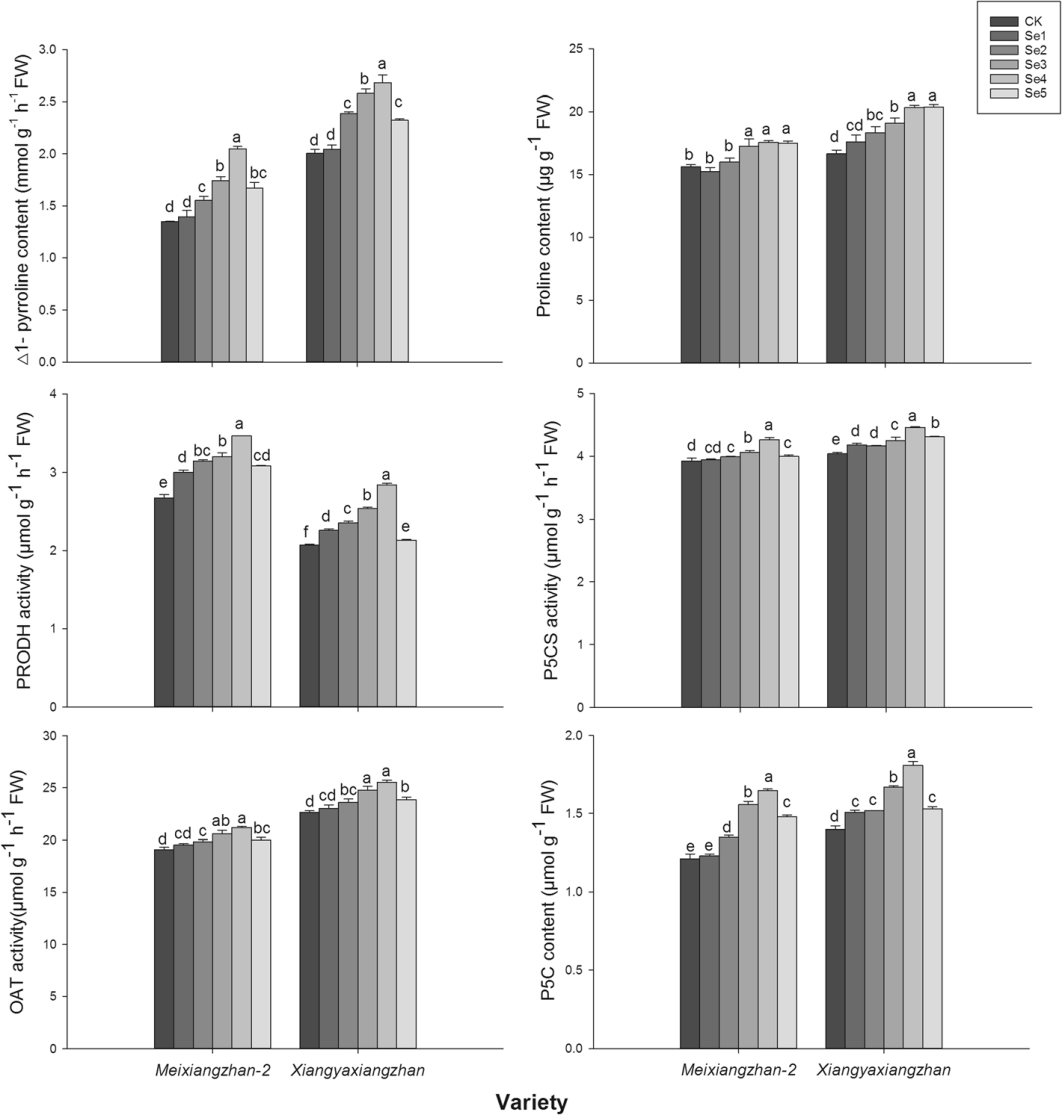
Effect of sodium selenate on the 2-AP synthetic precursor and activities of enzymes involved in 2-AP biosynthesis in grains

Foliar applications of sodium selenate affected 2-AP biosynthesis in terms of the △1-pyrroline, proline, PRODH, P5CS, P5C and OAT activities in grains (Fig. 3). Compared to CK, Se2, Se3, Se4 and Se5 significantly increased the △1- pyrroline content in grains, while the highest content was recorded in Se4 for both varieties. Higher grain proline contents were recorded in Se3, Se4 and Se5 than CK in both *Meixiangzhan-2* and *Xiangyaxiangzhan*. Compared to CK, Se treatments also significantly increased PRODH activity by 12.18–29.48% in *Meixiangzhan-2* and by 2.93–37.09% in *Xiangyaxiangzhan,* while the highest activity was recorded in Se4 for both varieties. In addition, the Se2, Se3, Se4 and Se5 treatments remarkably improved the P5C content, and the highest P5C content was recorded in Se4 for both varieties. Similar trends were observed for the OAT and P5CS activities.

### Correlation analysis between 2-AP and related precursors and enzymes

**Fig. 4:**
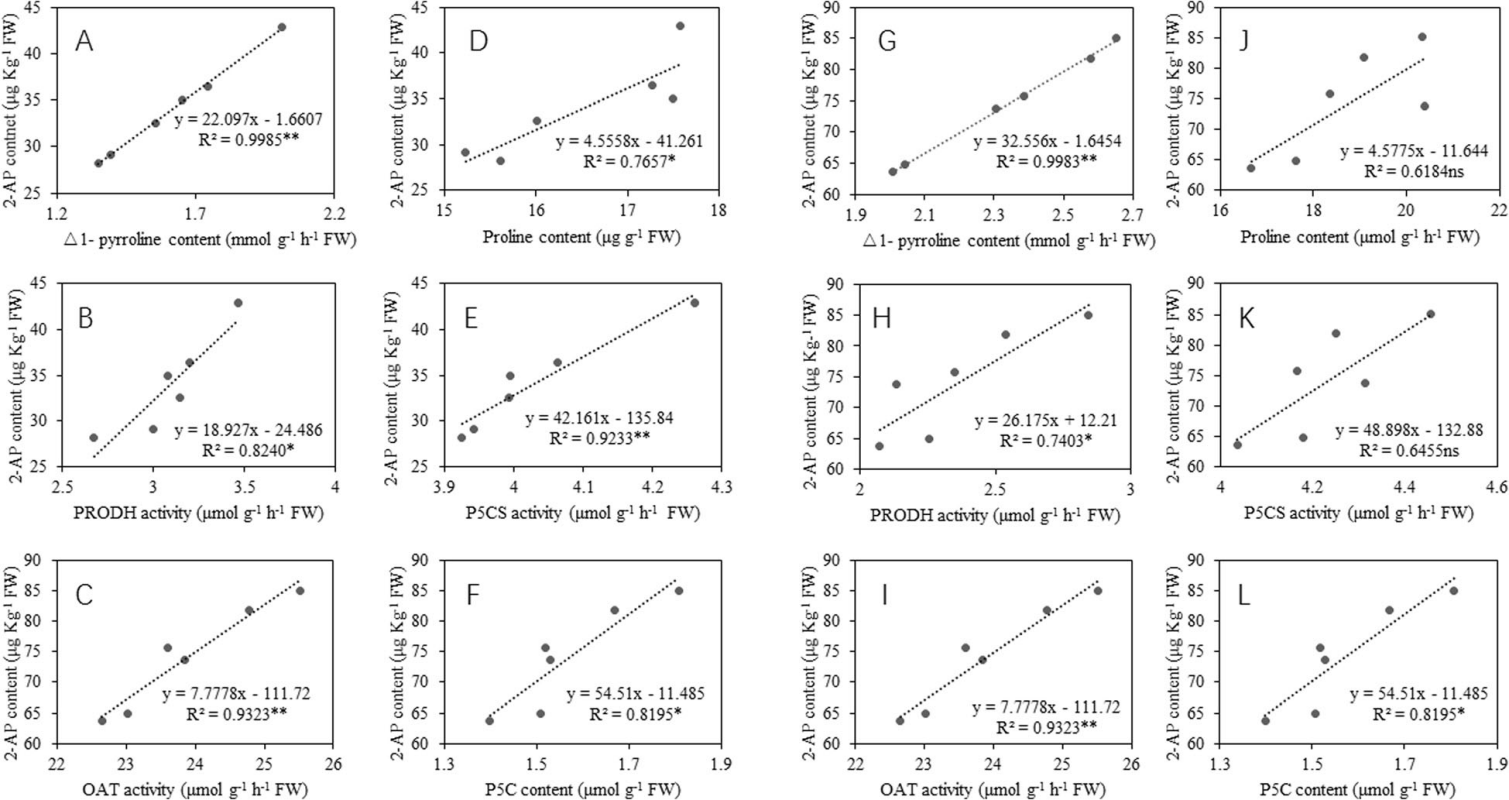
The correlation of 2-AP content in grains with precursors and enzymes involved in 2-AP biosynthesis for *Meixiang-2* (**a**-**f**) and *Xiangyaxiangzhan* (**g**-**l**)

As shown in Fig. 4, for *Meixiangzhan-2*, the △1- pyrroline content, proline content, PRODH activity, OAT activity, P5CS activity and P5C content all had a significant positive correlation with the 2-AP concentrations. For *Xiangyaxiangzhan*, there was a significant positive correlation between the 2-AP content and △1- pyrroline content. The 2-AP content in grains also had similar relationships with PRODH activity, OAT activity and P5C content. However, there was no significant correlation between the 2-AP content and proline content just like the 2-AP content and P5CS activity.

### MDA contents, osmo-protectants and antioxidant responses in leaves

**Fig. 5:**
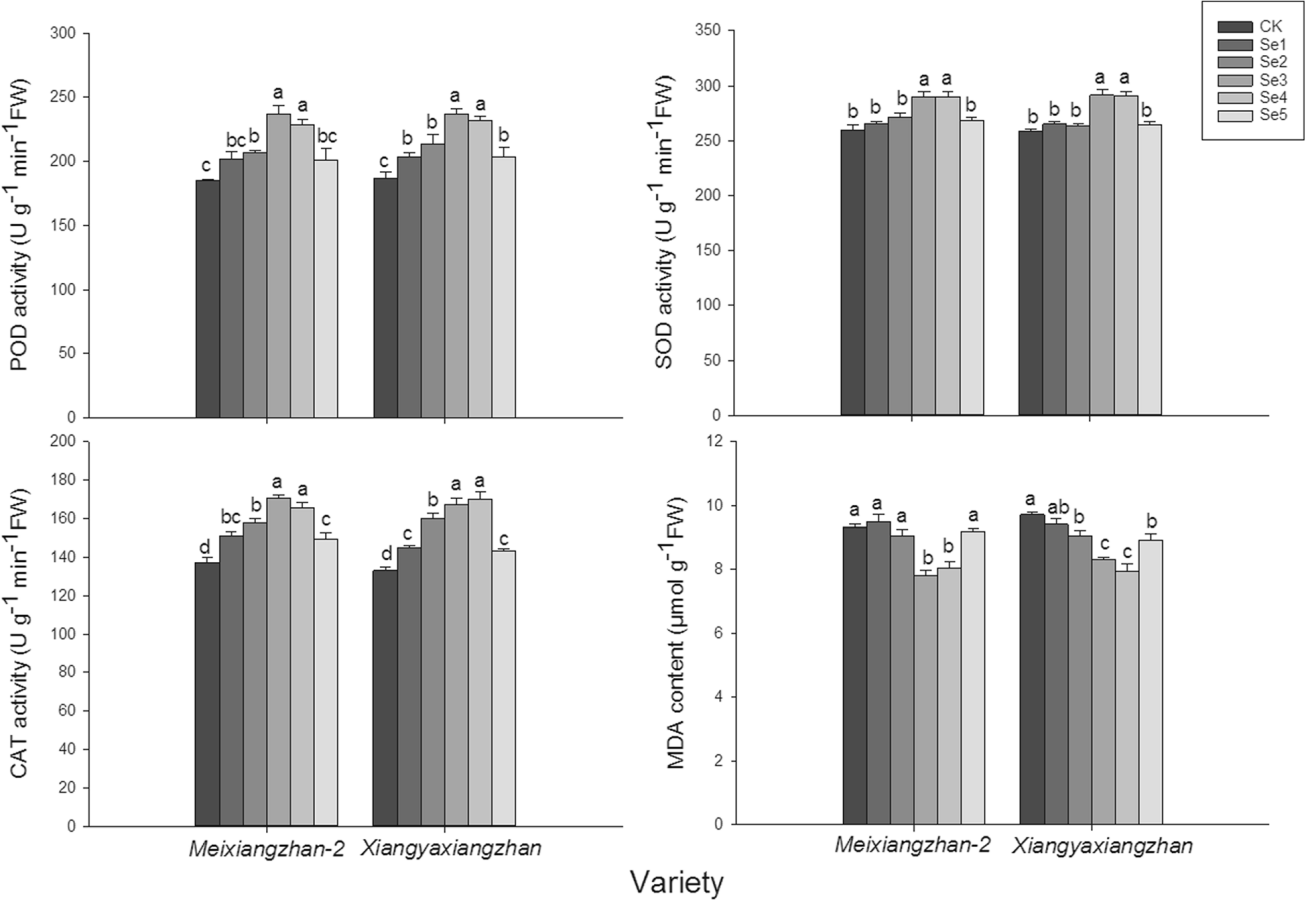
Effect of sodium selenate on POD, SOD, and CAT activities and the MDA content in leaves

Foliar applications of sodium selenate affected the antioxidative enzymatic activities in terms of SOD, POD and CAT (Fig. 5). Notably, 9.14, 11.62, 28.30, 23.70 and 8.72% higher POD activities were recorded in Se1, Se2, Se3, Se4 and Se5 than CK, for *Meixiangzhan-2,* while 8.58, 14.14, 26.60, 23.97 and 8.97% higher POD activities in Se1, Se2, Se3, Se4 and Se5 than CK for *Xiangyaxiangzhan*. There was no significant difference in the SOD activities among CK, Se1, Se2 and Se5, while SOD activity was significantly higher in Se3 and Se4 than in CK. CAT activities were ordered as follows: Se4 = Se3 > Se2 > = Se1 > = Se5 > CK for *Meixiangzhan-2* and Se4 = Se3 > Se2 > Se1 = Se5 > CK for *Xiangyaxiangzhan*. Furthermore, compared to CK, the Se3 and Se4 treatments significantly reduced the MDA content in leaves for both varieties.

### The expression of genes retaliated to antioxidative enzymatic activities

**Fig. 6:**
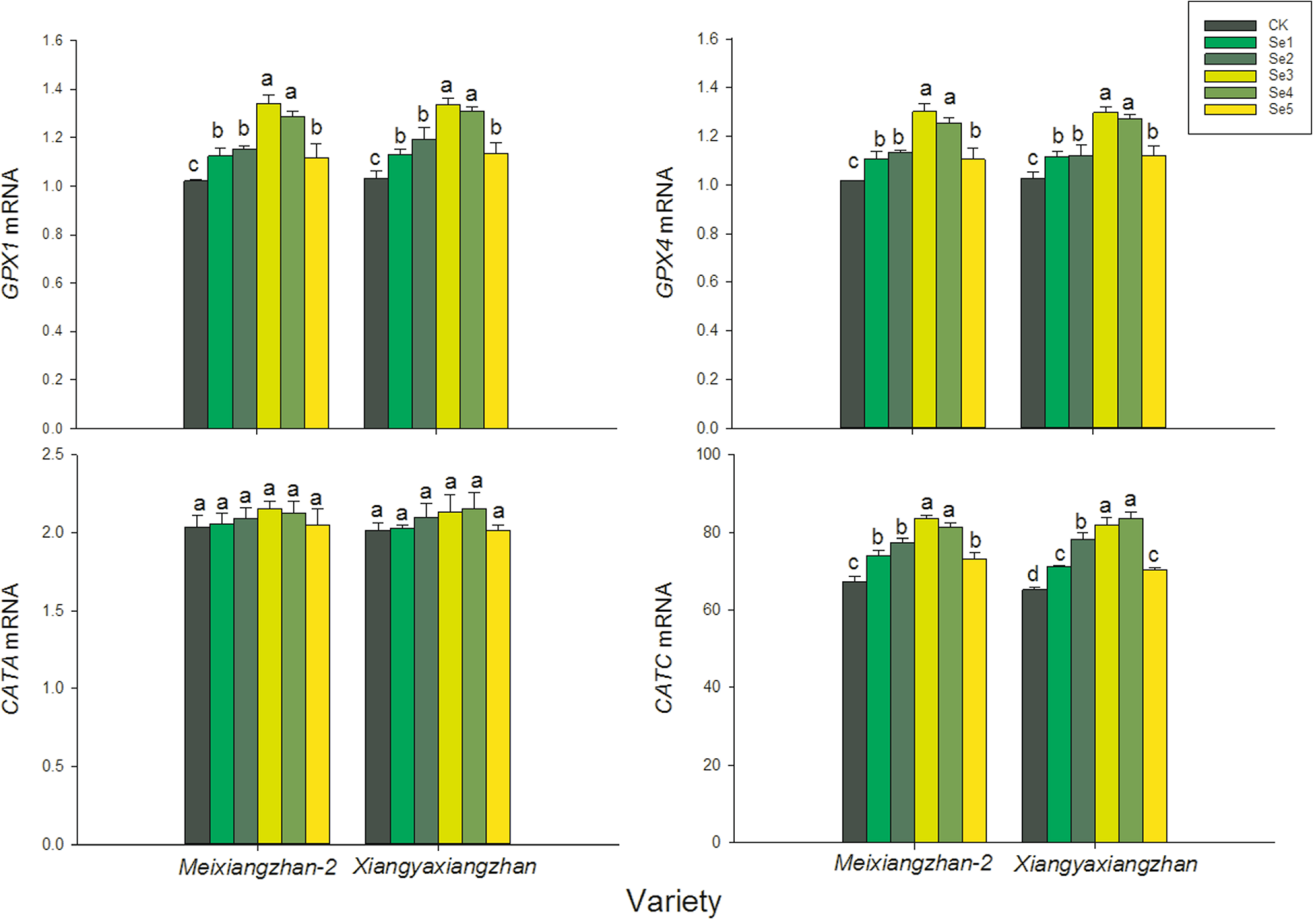
Analysis of transcript levels of *GPX1*, *GPX4*, *CATA* and *CATC*

Real-time PCR analyses depicted that levels of *GPX1*, *GPX4* and *CATC* transcripts were higher in in Se treatments (Fig. 6). Compared to CK, foliar applications of selenate significantly increased the expression of gene *GPX1*, *GPX4* and *CATC* genes by 9.64–31.28%, 8.75–28.12% and 7.63–27.85%, respectively. However, there was no remarkable difference among CK, Se1, Se2, Se3, Se4 and Se5 in *CATA* transcript.

### Se content in grains and leaves

**Fig. 7:**
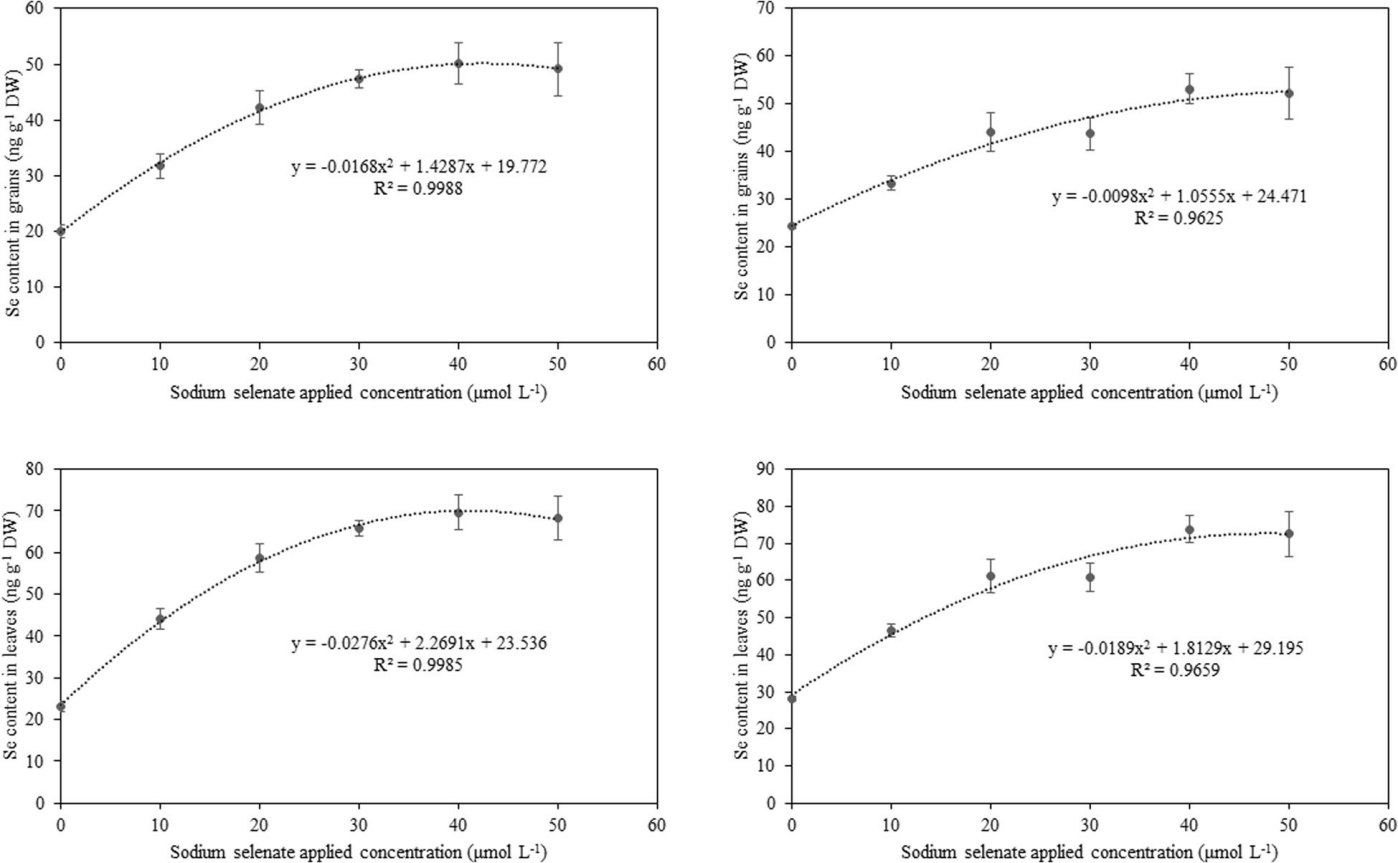
Effect of sodium selenite on Se content in leaves and grains in fragrant rice for *Meixiang-2* (**a**-**b**) and *Xiangyaxiangzhan* (**c**-**d**)

As shown in Fig. 7, foliar applications of sodium selenate significantly increased the Se content in leaves and grains for both fragrant rice varieties. For *Meixiangzhan-2*, compared to CK, Se1, Se2, Se3, Se4 and Se5 increased the Se content in grains by 58, 110, 136, 150 and 145%, respectively, and the Se content in leaves by 91, 154, 185, 202 and 196%, respectively. For *Xiangyaxiangzhan*, compared to CK, Se1, Se2, Se3, Se4 and Se5 increased the Se content in grains by 36, 80, 79, 117 and 113%, respectively, and the Se content in leaves by 65, 117, 116, 162, 158 and 196%, respectively.

### Grain yield

As shown in Table 1, for *Meixiangzhan-2*, there was no significant difference in the panicle number and grain number among CK, Se1, Se2, Se3 and Se5. However, compared to CK, the Se3 treatment significantly increased the seed-setting rate, while the 1000-grain weight demonstrated the following trend: Se3 > Se4 = Se2 = Se1 > Se5 = CK. A similar trend was also recorded in grain yield. For *Xiangyaxiangzhan*, higher seed-setting rates were recorded in Se2 and Se3 than in CK. The Se1, Se2, Se3 and Se4 treatments all significantly increased the 1000-grain weight and grain yield compared to CK, while the highest grain weight and yield were recorded in Se2 and Se3. Moreover, there was no significant difference among the CK, Se1, Se2, Se3 and Se5 treatments in either panicle number or grain number.

**Table 1:** Effect of sodium selenite on grain yield in fragrant rice

Variety	Treatment	Panicle number per hill	Grains number per panicle	Seed-setting rate (%)	1000-grain weight (g)	Grain yield (g pot^−1^)
Meixiangzhan-2						
	CK	10.60a	128.20a	77.55b	19.22c	61.00c
	Se1	10.06a	129.10a	78.09b	19.50b	62.22bc
	Se2	10.34a	126.81a	80.02ab	19.65b	64.33b
	Se3	10.35a	131.99a	83.07a	20.02a	68.11a
	Se4	10.73a	129.21a	79.88ab	19.65b	64.22b
	Se5	10.13a	127.79a	76.95b	19.33c	60.89c
Xiangyaxiangzhan						
	CK	10.58a	120.54a	76.25b	19.18c	55.56c
	Se1	10.52a	123.16a	78.89ab	19.54b	58.56b
	Se2	10.63a	121.27a	80.95a	19.99a	61.44a
	Se3	9.98a	122.95a	81.78a	19.79ab	61.43a
	Se4	10.48a	119.47a	79.17ab	19.54b	58.78b
	Se5	10.63a	122.09a	77.36b	18.88c	55.44c
Analysis of variance						
Selenium (Se)		ns	ns	**	**	**
Variety (V)		ns	**	ns	ns	**
Se × V		ns	ns	ns	*	*

Different letters indicate statistical significance at the *P* = 0.05 level for the same variety. Same as below

### Grain quality

As shown in Table 2, foliar applications of sodium selenate significantly affected the grain quality attributes of fragrant rice. For *Meixiangzhan-2*, a higher crude protein content was recorded in Se2, Se3 and Se4 than in CK. Compared to CK, Se2 and Se3 reduced the chalky rice rate and chalkiness significantly, and the lowest chalky rice rate and chalkiness were recorded in Se3. For *Xiangyaxiangzhan*, compared to CK, the Se1, Se2, Se3, Se4 and Se5 treatments all significantly improved the crude protein content. Low chalky rice rates were recorded in Se2 and Se3, while the lowest chalky rice rate was recorded in Se2. Moreover, there was no remarkable difference in the brown rice rate, mill rice rate and head rice rate between the CK and Se treatments for both varieties.

**Table 2:** Effect of sodium selenite on grain quality attributes in fragrant rice

Variety	Treatment	Brown rice rate (%)	Milled rice rate (%)	Head rice rate (%)	Crude protein content (%)	Chalky rice rate (%)	Chalkiness (%)
Meixiangzhan-2							
	CK	75.86a	66.95a	51.51a	8.51b	8.10a	2.04a
	Se1	76.88a	65.53a	52.42a	8.70ab	7.90a	1.98a
	Se2	75.79a	66.29a	52.13a	8.95a	6.66b	1.09b
	Se3	76.17a	65.22a	51.99a	8.96a	3.82c	0.62c
	Se4	77.41a	65.71a	51.20a	8.98a	8.28a	1.83a
	Se5	76.60a	65.71a	51.51a	8.66ab	7.79a	2.02a
Xiangyaxiangzhan							
	CK	75.82a	62.84a	52.49a	7.90c	1.87a	1.74a
	Se1	75.44a	62.20a	52.42a	8.23b	1.91a	1.68a
	Se2	75.14a	61.96a	52.45a	8.39ab	1.05c	1.71a
	Se3	75.20a	61.21a	52.73a	8.59a	1.41b	1.73a
	Se4	75.20a	61.60a	52.57a	8.52a	1.80a	1.89a
	Se5	74.91a	60.86a	52.37a	8.12b	1.74a	1.88a
Analysis of variance							
Selenium (Se)		ns	ns	**	**	**	**
Season (S)		*	**	**	**	**	*
Variety (V)		**	**	**	**	**	**
Se × S		ns	ns	ns	ns	ns	ns
Se × V		ns	ns	ns	ns	**	**
S × V		ns	ns	**	**	**	ns

## Discussion

The biosynthesis of 2-AP in fragrant rice is a complicated and important phenomenon which is influenced by several factors. Some researchers have conducted the experiments to study the effect of fertilizer and microelements on 2-AP content in fragrant rice. For example, the study of Ren [1] revealed that different water managements and nitrogen applications would significantly affect the contents of 2-AP and proline in aromatic rice. Li [12] demonstrated that Mn fertilizer could regulated the 2-AP content in fragrant rice grains by influencing some enzymes activities such as OAT and PRODH. Moreover, Bao [8] even revealed the molecular basis in enhancing the 2-AP contents under alternate wetting and drying environment in fragrant rice. Until now, there are two kinds of management might be able to enhance the 2-AP concentration in grains. One is to create some stress environments such as drought stress to stimulated the biosynthesis of proline which is the important precursors in 2-AP formation [10]. Another one is applied more nutrition elements such as nitrogen, silicon (Si) and Mn to promote the growth and development of fragrant rice and thus enhance the 2-AP biosynthesis [11, 18]. Furthermore, the study of Mo [19] even revealed that shading during the filling stage could improve 2-AP content in fragrant rice and indicated that there were some connections between the pathways leading to 2-AP and GABA production in fragrant rice.

Our study depicted the significantly effects of sodium selenate on 2-AP formation in fragrant rice. Compared to CK, most of Se applications increased the 2-AP content in grains and the highest content was recorded in Se4 treatment. This result could be explained by the enhancement of PRODH, P5CS and OAT activities which were related to 2-AP biosynthesis [20]. The significant positive correlation between 2-AP and activities of PRODH, P5CS and OAT was consistent with early study about 2-AP biosynthesis process in fragrant rice [9]. Furthermore, we observed that higher proline and △1-pyrroline contents in grains were recorded in Se treatments and there was also a significant positive correlation between △1-pyrroline content and 2-AP content. This result agreed with the study of Poonlaphdecha [21] who indicated that △1-pyrroline content was a limited factor in 2-AP biosynthesis in fragrant rice. Therefore, foliar applications of sodium selenate might be able to enhance the activities of some enzymes such as PRODH and OAT and thus promote the biosynthesis of 2-AP in fragrant rice.

Grain yield of fragrant rice was also influenced by Se applications. Some foliar applications of sodium selenate (Se2, Se3 and Se for *Meixiangzhan-2*, Se1, Se2, Se3 and Se4 for *Xiangyaxiangzhan*) increased fragrant grain yield remarkably. Those increment could be explained by the improvement in grain weight and seed-setting rate due to foliar application of sodium selenate. Our result agreed with the study of Yong [22] who demonstrated that foliar application of selenium fertilizer could increase yield and nutrients concentration of rice. Furthermore, grain quality is a determinant factor in economic returns for farmers and is normally evaluated by serval characteristics including milling, appearance and nutrient qualities [23]. Present study showed that foliar applications of sodium selenate regulated the fragrant rice grain quality in terms of appearance and nutrient qualities because of the increments in crude protein contents and decrement in chalky rice rates.

Moreover, present study showed that foliar application of sodium selenate regulated the antioxidant enzymatic activities at grain filling stage in terms of POD, CAT and SOD while decreasing the lipid per-oxidation (MDA concentration). POD, SOD and CAT were the key enzymes in quenching the reactive oxygen species and maintaining cellular structures and functions [23, 24]. The improvement in POD, SOD and CAT activities in present study agreed with the study of Diao [25] which revealed that selenium could promote the performance of tomato seedlings under salt stress by enhancing chloroplast antioxidant defense system. Similar result was also reported by Ríos [26] who found that application of selenite at low rate could induce higher increases in activities of enzymes that detoxify H_2_O_2_, especially glutathione (GSH) peroxidase and SOD. SOD, POD and CAT are key antioxidant enzymes which aid cells to remove the harmful oxygen species. The increment in antioxidative enzymatic activities might be attributed to the up-regulation of transcriptional expression of *GPX1*, *GPX4* and *CATC*. Previous study revealed that *GPX1* and *GPX4* are involved in biotic and abiotic stress responses and have the molecular function of regulating activities of antioxidant enzymes such as POD and SOD (GO:0004601) [27, 28]. The study of Zhang [29] discovered that the CAT enzyme pattern in rice leaves is similar to the *CATC* isoform enzyme expressed and made a hypothesis that the predominant CAT enzyme in rice leaves is a homo-oligomer consisting of *CATC* isoforms. The results of present study indicated that foliar of sodium selenate could enhance the fragrant rice stress resistance and help to ensure the stability of fragrant rice production.

In our study, higher contents of Se in grains were recorded in foliar application of selenate treatments. The grain Se contents in Se2, Se3, Se4 and Se5 treatments exceeded 40 μg kg^− 1^ which reached the standard of rich selenium paddy in China (GB/T 22499–2008). On the other hand, although the grain Se content increased with the increment of foliar application concentration, we observed that responses of fragrant rice to different selenate concentrations were different. The positive effects of foliar application of sodium selenate seemed to be diminishing at high applied concentration (Se5). Just like the other microelements, only the appropricate concentration of Se could bring the benefits to the plants. The study of *Khaliq* et al. [30] showed that high selenate levels inhibited germination of rice. Our study was consistent with the result of *Du* et al. [31] who demonstrated that low concentration of Se enhanced the growth and development of rice seedling while the high concentration would reduce the dry matter weight of seedling. Considered the yield performances, grain quality and 2-AP concentrations, the Se3 was the recommendation concentration in field practical application for fragrant rice. However, this recommendation might be different because of the soil type and plant species.

## Conclusion

Foliar applications of sodium selenate at initial heading stage can result in higher grain 2-AP concentration by enhancing the activities of PRODH, OAT and P5CS and increasing the content of proline, △1-pyrroline and P5C.The grain yield of fragrant rice increased due to selenate application just like grain protein content. The Se application also reduced the chalky rice rate and chalkiness. Furthermore, foliar applications of sodium selenate induced the regulation in the anti-oxidative enzymatic system in terms of SOD, POD, CAT activities and expression of gene *GPX4*, *GPX1* and *CATC*. The 30 μmol L^− 1^ sodium selenate was optimal applied concentration in present study.

## Methods

### Plant materials and growing conditions

Seeds of two fragrant rice varieties, ‘*Xiangyaxiangzhan*’ and ‘*Meixiangzhan-2*’, which are widely planted in Guangdong Province, China, were provided by the College of Agriculture, South China Agricultural University, Guangzhou China. A pot experiments during July to November in 2018 were conducted at the green house in Experimental Research Farm, College of Agriculture, South China Agricultural University, Guangzhou, China (23°16′ N, 113°23′ E and 11 m above the sea level). Each pot was filled with 9 kg of soil which was sandy loam with of 15.06 g/kg organic matter, 1.18 g/kg total nitrogen, 54.69 mg/kg available nitrogen, 1.16 g/kg total phosphorus, 18.06 mg/kg available phosphorus, 12.55 mg/kg total potassium, and 127.14 mg/kg available potassium, with a soil pH of 6.60. Seedlings of about 22-day-old were transplanted to the soil-filled pots with 5 hills per pot and 3 seedlings per hill. Each pot was applied with 1.9 g urea, 0.9 g phosphorus pentoxide and 0.9 g potassium oxide with 60% at basal and 40% at the tillering. The temperature of green house was between 23 and 29 °C during the experiment.

### Treatments and plant sampling

The six treatments were as follows: overhead sprinkling with 0, 10, 20, 30, 40 and 50 μmol·L^− 1^ sodium selenate at the initial heading stage; these treatments were known as CK, Se1, Se2, Se3, Se4 and Se5, respectively. On the15th days after the heading stage, fresh leaves and grains were separated and collected from the rice plants in each treatments, washed with double-distilled water and stored at − 80 °C for physio-biochemical analysis (grains were used for the determination of 2-AP synthetic precursors and enzymes involved in 2-AP biosynthesis; leaves were used for the determination of malondialdehyde (MDA), antioxidants, osmo-protectants and the qPCR). At maturity, fresh grains were separated and collected from the rice plants and stored at − 80 °C for 2-AP determination.

### Estimation of 2-acetyl-△1-pyrroline (2-AP) concentration in grains

The 2-AP concentration in grains was measured according to the methods of Du [7] by using the oscillations instrument (HZS-H, China) with a frequency of 200 oscillations per minute to treatment with the fresh grains. The 2-AP content was calculated after the synchronization distillation and extraction method (SDE) combined with GCMS-QP 2010 Plus (Shimadzu Corporation, Japan) and expressed as ug kg^− 1^.

### Measurements of △1-pyrroline, proline and pyrroline-5-carboxylic acid (P5C) content in grains

The grain △1-pyrroline content was determinate using the methods of Hill [32]. After the thirty minutes reaction with 1,4-diaminobutane at 30 °C, the content of △1-pyrroline was measured and after 30 min in room temperature. The content of proline in grains was measured with the methods of Bates [33]. The absorbance was read at 520 nm after the reaction with ninhydrin. The grain P5C content was determinate according to the method described by Wu [34]. After the reaction with trichloroacetic acid (TCA) and 2-aminobenzaldehyde, the absorbance was read at 440 nm.

### Determination of activity of proline dehydrogenase (PRODH), △△1-pyrroline-5-carboxylic acid synthetase (P5CS), ornithine aminotransferase (OAT) in grains

PRODH activity was measured according to the methods of Ncube [35]. The absorbance was read at 440 nm after reaction. The P5CS activity was determinate by the methods of Zhang [36]. 0.5 mL of enzyme extract was added into the reaction system contained 50 mM Tris-HCL buffer, 20.0 mM MgCl2, 50 mM sodium glutamate, 10 mM ATP, 100 mM hydroxamate-HCL. The activity of OAT was assayed following the methods of Chou [37]. After the reaction, the absorbance was read at 440 nm and the activity was calculated by extinction coefficient 2.68 mM^− 1^ cm^− 1^.

### Determination of malondialdehyde (MDA), anti-oxidants and osmo-protectants in leaves

The content of MDA and activities of POD, SOD and CAT were determined according to the methods described by Kong [38]. The content of MDA was measured by reacting with thiobarbituric acid (TBA) and the absorbance was read at the 532 nm, 600 nm, and 450 nm and the final result was expressed as μmol/g FW. The activity of peroxidase (POD EC1.11.1.7) was determined after the reaction which the solution was including enzyme extract, 0.3% H2O2, 0.2% guaiacol and 50 mM l^− 1^ sodium phosphate buffer (pH 7.0) and one POD unit of enzyme activity was expressed as U g^− 1^ min^− 1^ FW). The superoxide (SOD, EC 1.15.1.1) activity was measured by using nitro blue tetrazolium (NBT). The enzyme extract was added into the reaction mixture which contained sodium phosphatebuffer (pH 7.8), 130 mM methionine buffer, 750 μmol L^− 1^ NBT buffer, 100 μmol L^− 1^ EDTA-Na_2_ buffer and 20 μmol L^− 1^ lactoflavin. After reaction, the absorbance was read at 560 nm. One unit of SOD activity is equal to the volume of extract needed to cause 50% inhibition of the color reaction (U g-1 min-1 FW). Catalase (CAT, EC1.11.1.6) activity was determinate by adding an aliquot of enzyme extract to the reaction solution containing 0.3% H_2_O_2_ and sodium phosphate buffer. The the absorbance was read at 240 nm and CAT activity was expressed as U g^− 1^ min^− 1^ FW.

### Estimation of yield and its related traits

At maturity, six pots were randomly harvested from each treatment and threshed by machine. Then, the harvested grains were sun-dried and weighted in order to determinate the grain yield. Meanwhile, the rice plants of four pots from each treatment were collected respectively to estimate the average effective panicles number per hill, grain number per panicle, seed-setting rate and 1000-grain weight.

### Measurement of grain quality attributes

After sun drying, grains were stored at room temperature for at least a month to determine grain quality components. About 1.0 kg rice grains from each treatment was taken from storage and brown rice rate was estimated using a rice huller (Jiangsu, China) while milled rice and head rice recovery rates were calculated by using a Jingmi testing rice grader (Zhejiang, China). Grains with chalkiness and chalkiness degree were estimated by using an SDE-A light box (Guangzhou, China) while an Infratec-1241 grain analyzer (FOSS-TECATOR) was used to determine the protein contents.

### Real-time quantitative RT-PCR

Fresh leaves (0.03 g) were collected for total RNA extraction. Total RNA was extracted using HiPure Plant RNA Mini Kit (Magen, Guangzhou, China). The quality and quantity of RNA was assessed by Nanodrop 2000. The Hiscript II QRT SuperMix for qPCR (+gDNA wiper) (Vazyme, Nanjing, China) was used to synthesize cDNA from 500 ng of total RNA. The following mixtures were prepared in qPCR tubes: 4.4 μl cDNA, 0.2 μl each for forward and reverse primers, 5 μl 2*chamQ SYBR qPCR Master MiX and 0.2 μl ROX reference Dye 1, ddH2O to 20 μl (Vazyme, Nanjing, China). Real-time quantitative RTPCR (qRT-PCR) was conducted in CFX96 real-time PCR System (Bio-Rad, Hercules, CA, USA). Each RNA sample was performed in triplicate. A negative control without cDNA template was always included. Primers used for qRT-PCR were listed in Table 2. All primers were designed using the software tool Primer 5 (Table 3).

**Table 3:** Primer sequences of genes encoding enzymes involved in 2-AP synthesis in rice grains

Gene name	Accession No.	Primer sequences
*GPX1*	AK062772	F 5′-AGCAACCTGCACTTATGCACT-3′ R 5′-CAGCAAGGAAATTTATTGACATGA-3′
*GPX4*	AK243433	F 5′-CTGTACATATGCCTTGCCTCA-3′ R 5′-GTTACAGGGGCCAGATAAGC-3′
*CATA*	AK065094	F 5′-CGTCAACACCTACACCTTCG-3′ R 5′-CTCGTCGTCCATCAAGCAG-3′
*CATC*	AK062174	F: 5′-TGCCAAGGAGAACAACTTCA-3′ R: 5′- CCAGTAGGAGAGCCAGATGC-3′

### Statistical analysis

Statistix 8.1 (Analytical Software, Tallahassee, FL, USA) were used to analyze the experimental data while differences among means were separated by using least significant difference (LSD) test at 5% probability level. Graphical representation was conducted via Sigma Plot 14.0 (Systat Software Inc., California, USA).

## Data Availability

Not applicable.

## Abbreviations

2-AP: 2-acetyl-△1-pyrroline
CAT: catalase
MDA: malondialdehyde
OAT: ornithine aminotransferase
P5C: pyrroline-5-carboxylic acid
P5CS: △1-pyrroline-5-carboxylic acid synthetase
POD: peroxidase
PRODH: proline dehydrogenase
Se: Selenium
SOD: superoxide dismutase
